# Editorial: Myeloid-derived suppressor cells in inflammation and its complications and cancers

**DOI:** 10.3389/fimmu.2023.1240415

**Published:** 2023-07-18

**Authors:** Xing Li, Dinesh Kumar Ahirwar, Xiang-Yuan Wu

**Affiliations:** ^1^ Department of Medical Oncology and Guangdong Key Laboratory of Liver Disease Research, The Third Affiliated Hospital of Sun Yat-sen University, Guangzhou, China; ^2^ Department of Bioscience & Bioengineering, Indian Institute of Technology Jodhpur, Karwar, Rajasthan, India

**Keywords:** myeloid-derived suppressor cells, chronic inflammation, cancer, extramedullary erythropoiesis, erythroid progenitor cells

Obstruction of the differentiation and development of myeloid progenitor cells is a critical immune system disorder that occurs under physiological conditions including cancer, chronic or acute inflammations (1–5). In normal physiological conditions, common myeloid progenitor cells (CMP) differentiate into megakaryocyte erythroid progenitor cells (MEP) and granulocyte-monocyte progenitor (GMP) (6). When the process of GMP differentiation and development towards granulocytes and monocytes is blocked, the immature myeloid cells develop immune suppressive function without gaining proinflammatory capabilities. These cells were defined as myeloid-derived suppressor cell (MDSCs) (7, 8).

In this Research Topic focused on MDSC in chronic inflammation and cancer, several interesting studies investigated the roles of MDSCs in pathologic progression from inflammation to malignancies. While immune suppressive function is a basic feature of MDSCs (8, 9), their roles in chronic inflammatory diseases are controversial (1). In different conditions, MDSC may either promote or relieve chronic diseases. Our previous studies found that MDSC relieved chronic inflammatory diseases, including asthma and neonatal necrotizing enterocolitis (10–12). *In vitro* generated MDSC by lactoferrin have also been shown to relieve bleomycin induced interstitial pneumonia (11). However, Liu et al. reported that idiopathic pulmonary fibrosis (IPF) related MDSC actually promoted IPF by activating lung fibroblasts and myofibroblast differentiation. Soluble B7H3 generated by lung fibroblasts induced and chemoattracted monocytic MDSCs (M-MDSCs) to IPF lesions, which in turn activated lung fibroblasts and myofibroblast differentiation. The mechanism of B7H3-dependent MDSCs in IPF displayed a disease-specific manner, indicating that glucocorticoids suppressed the level of M-MDSC. However, glucocorticoids have been reported to induce MDSC in other conditions (11).

In addition to activating myofibroblast differentiation in IPF, MDSCc aggravate Helicobacter-induced malignancies. Schlafen4^+^ MDSCs have been found to be a disease-specific MDSC in Helicobacter-induced gastric metaplasia and malignancies (13–17) by Ding et al. In this Research Topic, they revealed the role of GTPases in Schlafen4^+^ MDSCs. They found that disruption of Slfn4 or pharmacologic inhibition of PED5/6 after Helicobacter infection suppressed MDSC function and mitigated development of spasmolytic polypeptide-expressing metaplasia (SPEM). The role of MDSC in peritoneal metastases from gastric cancer was evaluated by Takahashi et al. They found that M2-type macrophages were significantly higher in patients with peritoneal metastases. CD14^+^ monocyte populations displayed different markers between patients with or without peritoneal metastases. Their results indicated the potential role of M-MDSC in peritoneal metastases.

Not only the pathological role of MDSCs, but their prognostic value is also under active investigation for multiple diseases (18, 19). Petrova et al. found that melanoma patients without visible metastasis were characterized by the absence of MDSC immunosuppressive activity. MDSC frequency was significantly increased in non-responders to immune checkpoint inhibitors (ICIs) treatment compared to responders. However, the methodology for studying MDSCs are not easily standardized. Previous studies have found that human MDSCs need to be studied on fresh PBMC. G-MDSC can be studied with delay, but M-MDSC should be studied no later than 4 h after blood draw (20). The quality control of T cell suppressive function experiments is even more strict, according to our experience.

Modulate the immune suppressive function of MDSC has been investigated to relieve MDSC mediated pathologic progress (10–12). Our studies found lactoferrin induced MDSC relieved inflammatory diseases (10–12). Multiple MDSC eliminating methodology were developed as well (21). Kaul et al. found that slit2 induced M1 macrophages to enhances antitumor immunity, which could also be a potential method to suppress the development or suppressive function of MDSC.

In the last decade, extramedullary erythropoiesis has been found in the spleen in cases of anaemia, inflammation, and tumours, with a large number of CD71^+^erythroid progenitor cells (EPCs) accumulated (3–5, 22). There are two subtypes of CD71^+^EPCs. CD45^+^CD71^+^EPCs present immune suppressive function, which limits anti-infection immunity and promotes tumour development (3, 5, 23). CD45^-^EPCs promote tumour progression through secreting artemin (22). In 2022, a study found mixed development of erythroid and myeloid differentiation in the background of tumours. They found that EPCs had the potential to differentiate into myeloid cells, which was defined as erythroid-differentiated myeloid cells (EDMCs) (24), indicating that trans-differentiation of CMP progeny cells was a critical immune system disorders in the background of cancer. Our study found that CD45^+^EPCs were involved in chronic hepatitis B (CHB) (4) and CHB associated hepatocellular carcinoma (HCC) (23). CD45^+^EPCs inhibited HBsAg seroclearance during finite pegylated interferon treatment through LAG3 and TGF-β (4). CHB related CD45^+^EPCs presented myeloid cell morphology as well (4). Besides, EPCs, instead of MDSC, were the major immune suppressive cells in HCC microenvironment (23). Thus, our study indicated that EDMCs might be involved in the transformation of CHB to HCC. In summary, obstruction of the differentiation and development of myeloid progenitor cells lead to accumulation of MDSC and EPC, as well as EDMCs, which plays a critical role in inflammation, cancer and their transformation.

In conclusion, this Research Topic provides a comprehensive overview of the current understanding of MDSC in chronic inflammation and cancer. Although the roles of MDSCs in different diseases varied and is sometimes controversial, targeting MDSC might be a promising therapeutic strategy for multiple diseases, especial chronic inflammatory diseases and cancer. The mechanism underlies the obstruction of the differentiation and development of myeloid progenitor cells was disease specific and needs further investigation.

## Author contributions

All authors listed have made a substantial, direct, and intellectual contribution to the work and approved it for publication.
